# Light sheet fluorescence microscopy for the investigation of blood-sucking arthropods dyed via artificial membrane feeding

**DOI:** 10.1186/s13071-022-05157-2

**Published:** 2022-02-12

**Authors:** Lars ten Bosch, Birgit Habedank, Alessia Candeo, Andrea Bassi, Gianluca Valentini, Christoph Gerhard

**Affiliations:** 1 Faculty of Engineering and Health, University of Applied Sciences and Arts, 37085 Göttingen, Germany; 2 Section Health Pests and Their Control, German Environment Agency, Corrensplatz 1, 14195 Berlin, Germany; 3 Dipartimento di Fisica, Politecnico di Milano, Piazza Leonardo da Vinci 32, 20133 Milan, Italy; 4 Istituto di Fotonica e Nanotecnologie, Consiglio Nazionale delle Ricerche, Piazza Leonardo da Vinci 32, 20133 Milan, Italy

**Keywords:** Light sheet fluorescence microscopy, *Pediculus humanus*, Haematophagous arthropods, Blood, In vivo, Artificial membrane feeding

## Abstract

Physical methods to control pest arthropods are increasing in importance, but detailed knowledge of the effects of some of these methods on the target organisms is lacking. The aim of this study was to use light sheet fluorescence microscopy (LSFM) in anatomical studies of blood-sucking arthropods in vivo to assess the suitability of this method to investigate the morphological structures of arthropods and changes in these structures over time, using the human louse *Pediculus humanus* (Phthiraptera: Pediculidae) as sample organism. Plasma treatment was used as an example of a procedure employed to control arthropods. The lice were prepared using an artificial membrane feeding method involving the ingestion of human blood alone and human blood with an added fluorescent dye in vitro. It was shown that such staining leads to a notable enhancement of the imaging contrast with respect to unstained whole lice and internal organs that can normally not be viewed by transmission microscopy but which become visible by this approach. Some lice were subjected to plasma treatment to inflict damage to the organisms, which were then compared to untreated lice. Using LSFM, a change in morphology due to plasma treatment was observed.

These results demonstrate that fluorescence staining coupled with LSFM represents a powerful and straightforward method enabling the investigation of the morphology—including anatomy—of blood-sucking lice and other arthropods.

**Graphical Abstract:**

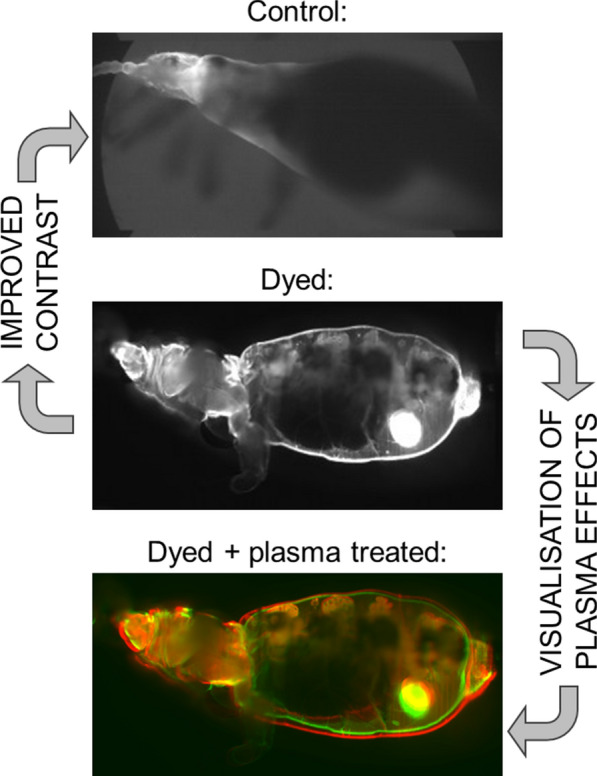

Until the second half of the twentieth century the most common optical method used for morphological studies on smaller arthropods was light microscopy. Light microscopy enabled visualisation of the external and internal structures of these organisms, although to study internal anatomical features, histological treatment of prepared sections and dissections of the arthropod were required. Up until the time adequate techniques were developed to photograph the morphological details in sufficient quality, the findings of such studies were illustrated as schematics hand-drawn by the researcher, a procedure requiring artistic skills and time. In recent decades, there has been a huge proliferation of new technologies that have improved the study of insect morphology, such as scanning electron microscopy (SEM), transmission electron microscopy (TEM), serial block-face SEM, focused ion beam (FIB), computer tomography, among others [1]. At the same time, biological imaging has seen a rapid expansion of the portfolio of available techniques for the study of morphology and development based on optics. In particular, the development and optimisation of optical sectioning techniques together with the design of new fluorescent probes have been a breakthrough. Widefield microscopy, one of most basic microscopy techniques, works well for thin sections of specimens, but in intact, three-dimensional (3D) samples the contrast is strongly reduced by out-of-focus light. Being able to optically section a sample—i.e. without the need for the fixation, cutting and embedding steps required by the histology process—has allowed researchers to visualise entire organs and organisms in 3D with easier or no sample preparation, with high contrast and often in vivo. Traditionally, the best known techniques employing optical sectioning are confocal laser scanning microscopy (CLSM), spinning disc microscopy and two-photon excitation microscopy. However, light sheet fluorescence microscopy (LSFM), also known as single plane illumination microscopy (SPIM), is increasingly becoming the technique of choice for the study of larger samples.

In LSFM, the specimen is illuminated laterally with a sheet of light and the fluorescence from a whole two-dimensional (2D) plane is collected orthogonally to the excitation axis and recorded by a camera. By moving the sample through the light sheet, various images of the sample are acquired at different depths, and these are then reconstructed digitally in 3D. Compared to other optical techniques mentioned above, LSFM is considered to be less aggressive due to the use of a lower dose of light that illuminates only the in-focus plane, and to be faster because of the use of fast cameras that capture a 2D plane in one shot. Moreover, the recorded field of view is large, while the resolution can be even subcellular. In addition, the orthogonal arrangement of the illumination and detection leaves space for a less invasive mounting of the sample so that the latter can be maintained alive in quasi-physiological conditions with minor impairment for a limited length of time after embedding. These characteristics have driven the integration of LSFM into developmental biology, particularly in long-term in vivo studies [2]. For example, LSFM has been successfully used for in vivo studies of plants, such as thale cress *Arabidopsis thaliana* [3], invertebrates, like the fruit fly *Drosophila melanogaster* [1, 4], the red flour beetle *Tribolium castaneum* [5] and the fresh-water polyp *Hydra vulgaris* [6], and vertebrates, such as the zebrafish *Danio rerio* [7, 8], the Japanese rice fish *Oryzias latipes* [9] and even a mouse embryo [10].

The morphology of haematophagous arthropods, including mosquitoes, ticks, mites, fleas and lice, such as, for example, the human louse *Pediculus humanus*, is of particular relevance in medical and veterinary sciences. An increasing number of studies on the toxicology, evolution and genetics of hematophagous insects, especially lice, are appearing in the literature [11–18]. However, the well-known sketches of *Pediculus humanus* prepared by Ferris in 1951 [19] are still among the most common literature sources for anatomical insights into the morphology of an entire human louse. Recently, single internal structures of comparable organisms have been visualised using SEM, FIB and CLSM [20, 21], leading to a partial characterisation of the parasite’s anatomy. The ultimate aim is to better understand how lice detect their hosts and to verify the efficacy of treatments against head lice.

In the work reported here, we applied LSFM, to our knowledge for the first time, to the in vivo study of *Pediculus humanus*, specifically the ecotype body louse (*Pediculus humanus humanus*), and analysed the efficacy of this method with that of a physical method used to control this parasite. Many novel non-pharmaceutical products for head lice control have been introduced on the market in recent years, based on various modes of action (mechanical, thermal or other). Here we assess the damage caused by in vivo plasma treatment to lice. LSFM was chosen as the technique of study due to its high speed, which should allow the avoidance of motion artefacts, gentleness, which enables the lice to be kept alive for several hours while not interfering with the plasma treatment and wide field of view, which will allow the study of a large portion of the lice. To demonstrate whether a physical external treatment gives rise to detectable changes in morphology, the lice were treated by a cold atmospheric pressure plasma; the setup used is custom-made, is based on the OpenSPIM approach and its schematic is shown in Fig. 1a [2, 22, 23].

**Fig. 1:**
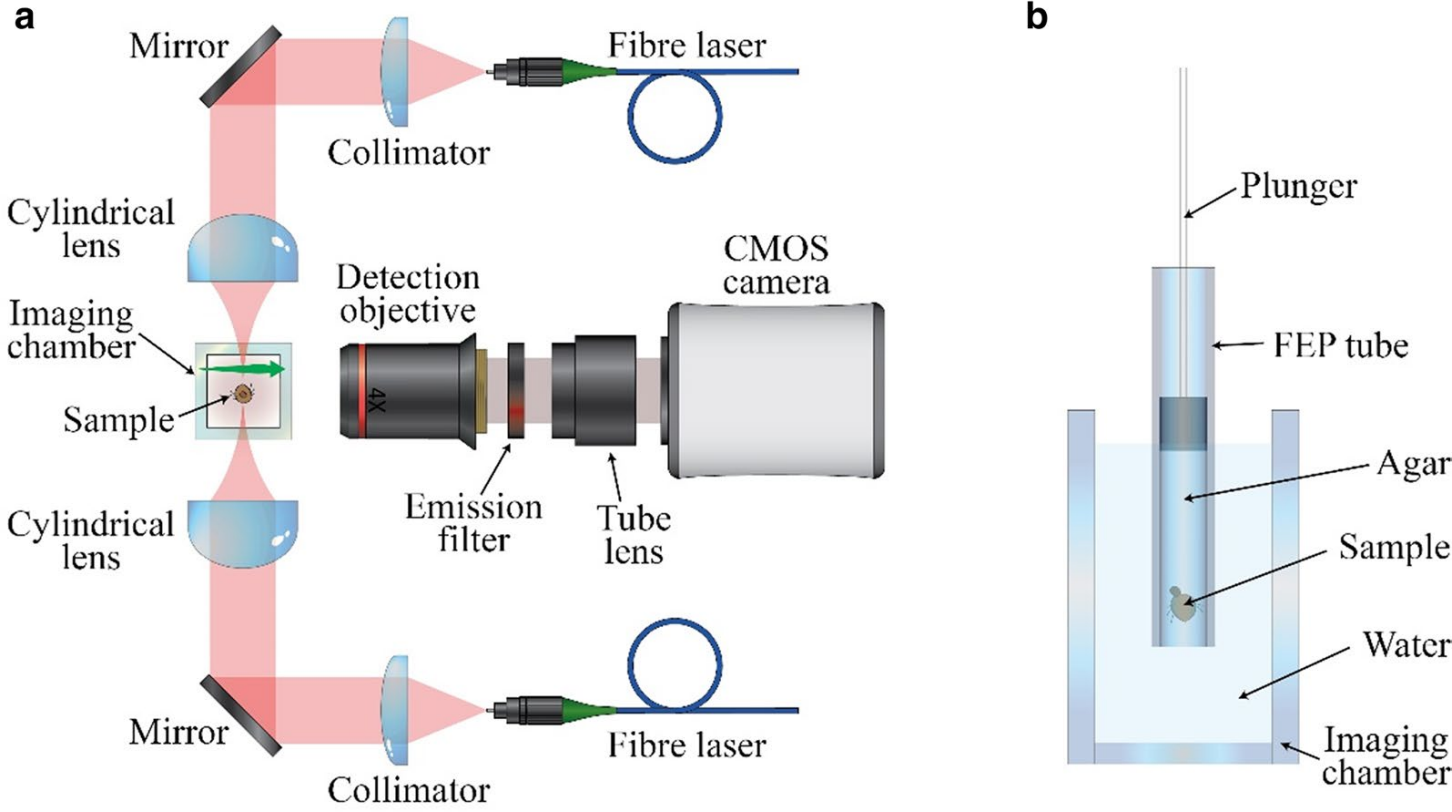
Schematic of the used light sheet fluorescence microscopy set-up (**a**) and of the imaging chamber with the sample embedded in agarose and held in place by a FEP tube (**b**). Abbreviations: CMOS, Complementary metal-oxide-semiconductor; FEP, fluorinated ethylene propylene

Specifically, the LSFM set-up consisted of a laser with a central wavelength of 647 nm (iBeam; TOPTICA Photonics AG, Munich, Germany), which is used to illuminate the sample. The laser light is fibre coupled and split in two branches with a fibre splitter. The two beams are then separately collimated to a 5-mm Gaussian waist by a collimator and focussed by cylindrical lenses (*f* = 75 mm) to create two counter-propagating light sheets, in a manner similar to that described in [24]. This approach is known as double-sided illumination, and the aim is to reduce shadowing artefacts due to absorbing elements in the sample and to maximise the penetration of the laser from both sides of the sample. A 4X (Nikon Europe BV, Amsterdam, Netherlands) long working distance microscope objective lens (numerical aperature = 0.13) is used to collect the fluorescence emitted orthogonally to the light sheet, in combination with an emission filter (bandwidth: 670–710 nm). A complementary metal-oxide-semiconductor (CMOS) camera (ORCA-Flash4.0; Hamamatsu Photonics K.K., Hamamatsu, Japan) is used to collect the images from an approximately 3 × 3-mm^2^ field of view. To acquire sequential images at various depths, the sample is moved through the light sheet with a translation stage, as indicated by the green arrow in Fig. 1a.

In this study, the tested organisms were the insecticide-sensitive body lice *Pediculus humanus humanus* that derived from the rabbit-adapted strain of the German Environment Agency (UBA, Berlin, Germany). This lice strain is reared at the UBA for investigations on the efficacy of products and procedures to control human lice. Body lice were chosen as a model for blood-sucking arthropods since even adult specimens feature sufficient translucency for the excitation and detection of fluorescence signals in depth within the organism, leading to adequate features contrast and good visibility of inner organs.

For the methodological investigation using LSFM, the lice were reared as previously reported for breeding an in vitro strain of *Pediculus humanus humanus* [25]. The lice, beginning at the juvenile stage, were fed in vitro using a Parafilm M membrane and fresh human blood of a volunteer donor until the instars developed into adults. The adult lice were then stored under constant conditions at 32 °C and 50% humidity until 12 h before the experiment. The specimens were fed on fresh human blood by exposing them to the in vitro feeding system for 30 min prior to LSFM analysis. The measurement was performed directly after feeding.

For image acquisition, the investigated lice were embedded in a low-melting-point agarose gel, which was then inserted into a fluorinated ethylene propylene (FEP) tube (FT2X3; Adtech Polymer Engineering Ltd., Stroud, UK) with the help of a plunger. The tube was then placed in the imaging chamber, consisting of a glass cuvette filled with water, as shown in Fig. 1b. Three different preparation methods were tested in sequential order:

(i) A control group of lice was embedded and introduced into the FEP tubing without any further treatment.
(ii) To increase the contrast of the images, in the second group of lice, stain (dye Nile blue; Nile blue 690 Perchlorate; CAS No.: 53340-16-2; Exiton Inc., Dayton, OH, United States) was added to blood fed to a group of lice and used as a contrast medium. The procedure consisted of dissolving the dye in ethylene glycol and then adding the solution to the feeding blood to obtain a target concentration of 0.4 mg/ml blood. Subsequent feeding was performed as in the first group.
(iii) To the last group of experimental lice, a group of lice already fed with stained blood was subjected to plasma treatment by directly inserting the lice into the filamentary discharge area of a cold atmospheric pressure plasma comb. This treatment procedure has been described in previous reports [26, 27], with a high efficiency against different developmental stages of the model organism *Pediculus humanus humanus* observed.

Two plasma treatments, with a duration of 1 and 5 s, respectively, were tested. Such treatments were carried out in order to assess the capability of LSFM to give first insights into possible plasma-induced modifications of the lice’s morphology. To prevent motion artefacts in the final image stacks, LSFM was performed at high speed (12 frames/s) at an excitation power of approximately 2 mW.

First, LSFM scans were performed on the control group of freshly fed lice, without adding dye to the human blood. As shown in Figs. 2 and  3a, the detected image presented an autofluorescence signal that led to good visibility of external morphological structures and accentuation of sclerotized structures, and a partial or poor contrast of the internal organs of the samples.

**Fig. 2:**
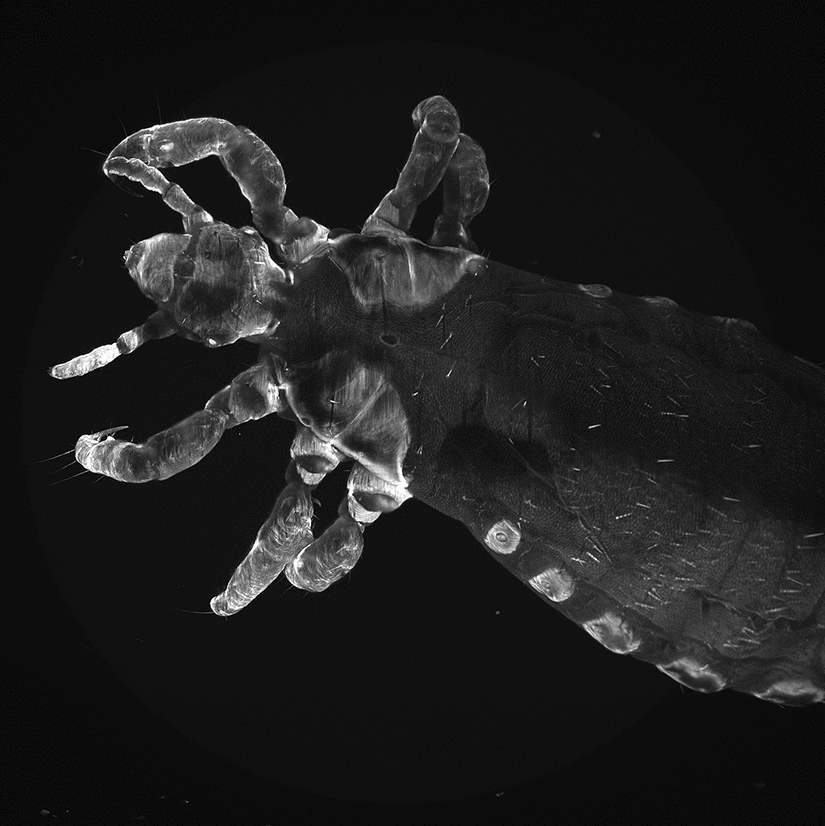
*Pediculus humanus humanus*, dorsal view (field of view 3 × 3 mm^2^). External morphological structures are visible, with accentuation to more sclerotized surface structures (e.g. on the head: labrum, clypeus, ocular-antennal segment, segments of antennae; segments of thorax and legs; paratergal plates of the abdomen). No internal structures are visible. The image shown is a maximum intensity projection of the acquired stack

**Fig. 3:**
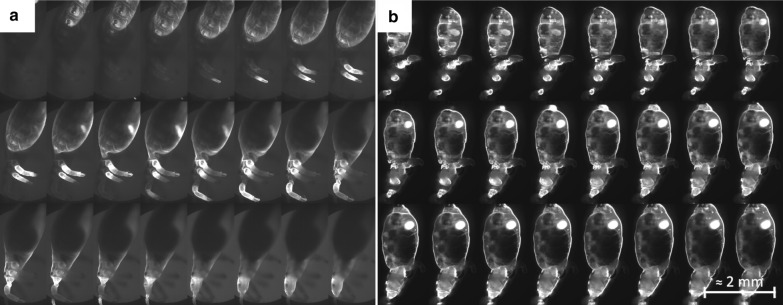
Montage of a sub-set of the images acquired at different depths with the light sheet fluorescence microscopy set-up for the lice groups without (**a**) and with (**b**) the contrast medium. While in **a** internal organs cannot be seen, in **b** internal organs can be clearly identified. For more detail, see Fig. 4

To achieve an acceptable contrast for the analysis of the internal organs of the samples, dye-enriched blood was fed to the lice as explained in preceding text. The sections acquired for the stained and unstained samples are presented for comparison in Fig. 3.

Each slice of the section of an undyed louse shows inadequate contrast, resulting in the internal organs being barely visible. However, in the stained louse, the fluorescence signal and the contrast are significantly enhanced. In this case, the image quality is sufficient to observed organs and morphological details in each scanned plane within the sample, as shown in Fig. 4. The dye spread rapidly throughout the organism, leading to high contrast at organ interfaces. Thanks to the high-speed acquisition (12 frames/s), no significant motion artefact or blur occurred due to peristaltic movement of the intestines. Moreover, embedding the sample within the agarose supresses the movement of the extremities. Consequently, only bowel movements become visible, shown as time-dependent variations within the pictures. A sagittal section of a dyed male adult is shown in Fig. 4. In addition to external structures, in the lateral view (Fig. 4a) several details can be identified: visible parts of the male’s reproductive system (testes, vesicula seminalis), parts of the digestive system (salivary glands, midgut and hindgut), parts of the excretory system (Malpighian tubules) and parts of the respiratory system (spiracles, tracheal system).

**Fig. 4:**
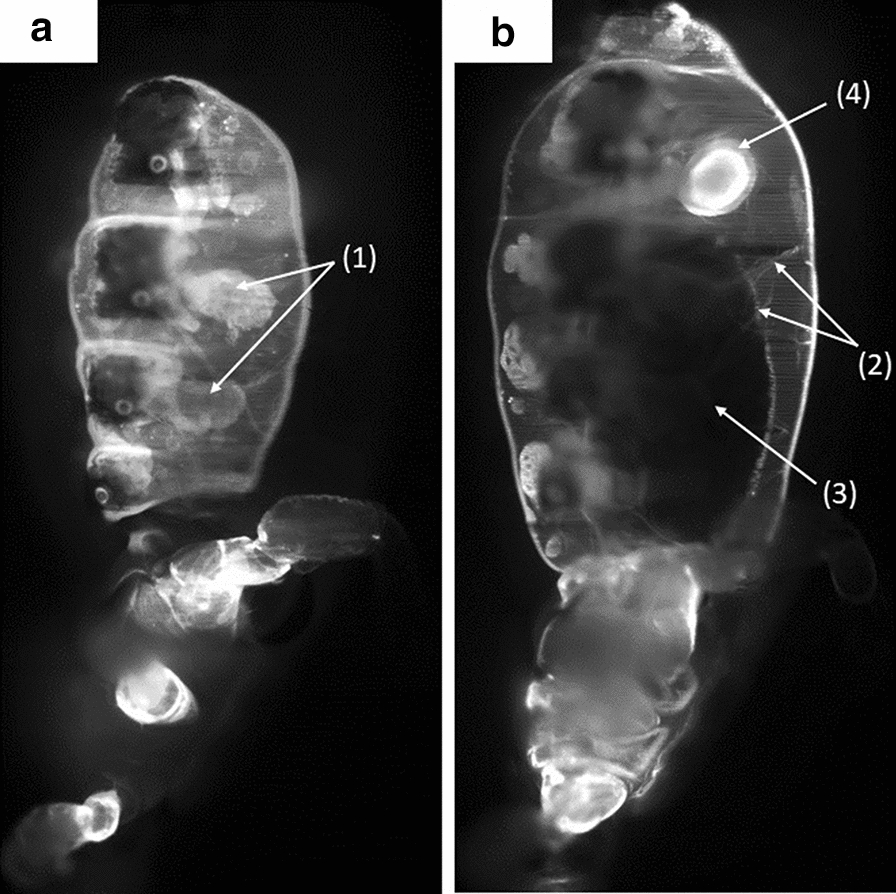
Detailed views of different illuminated sections of a stained male adult louse measured using the light sheet fluorescence microscopy set-up. **a** Near-surface section, with* (1)* indicating the testes of the individual. **b** Section close to the medial plane, with* (2)* indicating some of the tracheae in the abdomen,* (3)* indicating the blood-filled midgut,* (4)* indicating the *vesicula seminalis*

Based on the measurements collected from the experiments, the optical penetration and observation depth into the investigated lice were approximately 60% of the total thickness of the louse. This value was estimated taking the known thickness (approx. 2 µm) and number of single slices detected with LSFM into account. Several factors are known to limit the penetration depth and contrast. First, the effect of quenching can cause a notable decrease in fluorescence intensity; this especially applies to compartments with high concentrations of fluorescent dyes where self-quenching may occur. In this set-up, a large fraction of the emitted photons could be absorbed back by the fluorophore, which may lead to the selective suppression of fluorescence signals from regions or the intestines with a high blood volume and explain the dark shadow observed in the centre of Fig. 4b. Second, some areas can feature high scattering and absorption, leading to blurring and attenuation of both the incident excitation light and the resulting fluorescence signal. Third, a degradation of the dye over time can also be an issue, especially for longer time-lapse scans of the organism.

The analysis of global structural changes induced in the dyed lice upon application of the plasma treatment is shown in Fig. 5. A reference acquisition was performed directly after plasma treatments of 1 s and 5 s duration, respectively, and is colour-coded green. Subsequent images at different time (*t*_obs_) points were acquired without moving the sample from the imaging chamber (see Fig. 5). These images were colour-coded red and positioned to overlay the green reference images. A misalignment between the green and the red image indicates a variation with time. We observed that the visible area of treated lice increases over time. When the plasma treatment was applied for 1 s, almost no change was observed at an observation time of 15 min after treatment, while a very small change was observed at 120 min after treatment. However, when the plasma treatment was applied for 5 s, a measurable increase in area was observed 15 min after treatment, becoming relevant at 55 min after treatment.

**Fig. 5:**
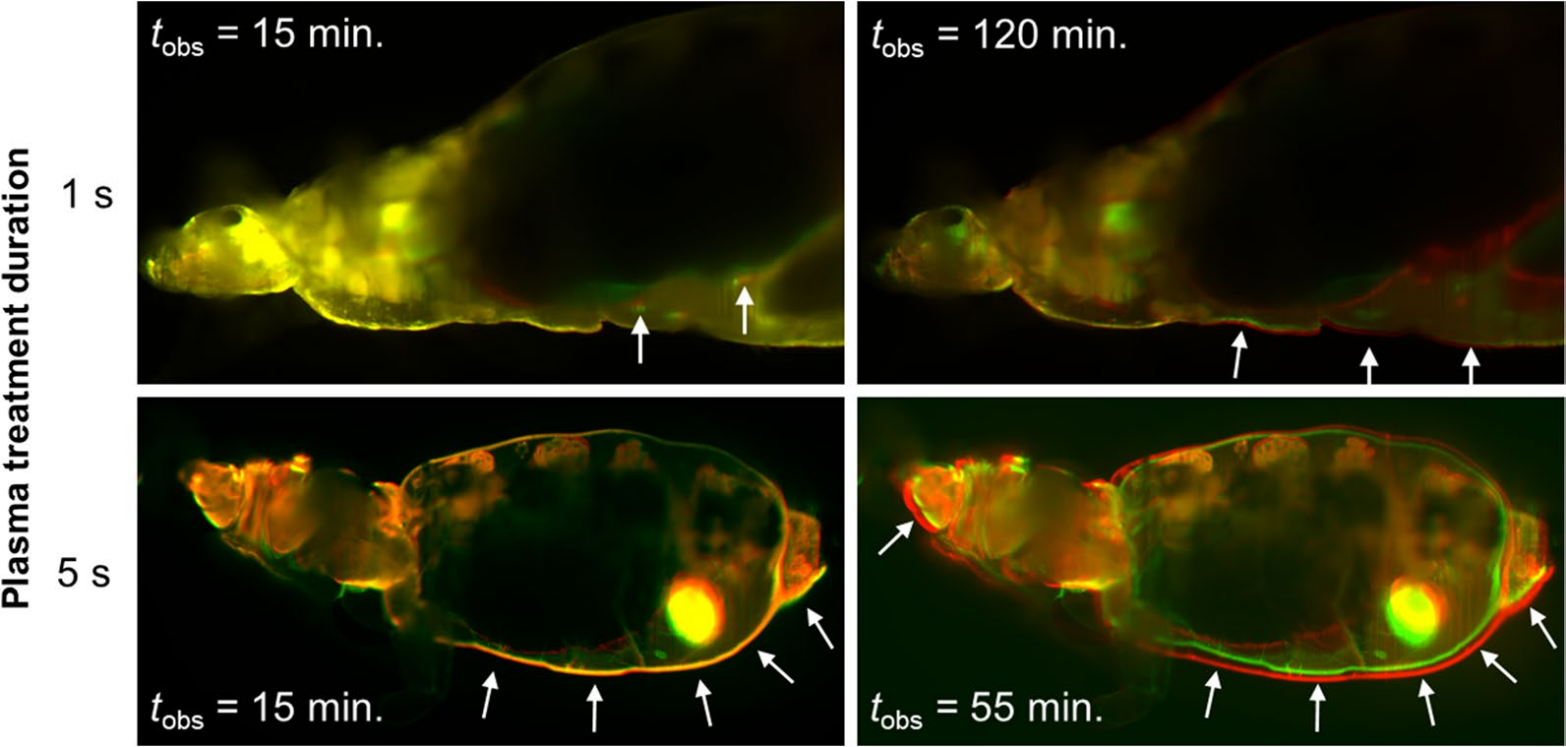
Comparison of lice plasma subjected to plasma treatment for different lengths of time (1 s and 5 s, respectively) and measured after different observation times (*t*_obs_). The four pictures are of two overlaid images acquired at the same sample depth. The green images were taken directly after introducing the sample into the imaging chamber. The red slices were taken after the treatment and at the specific observation time mentioned in the corresponding figure. The arrows indicate the areas of highest swelling observed over time

The LSFM analysis shows a correlation between the length of the plasma treatment and the resulting change in detectable area, thus demonstrating that the body swelling of the louse could be a direct effect of plasma. To quantify this observation, further studies with a higher number of individuals will be conducted in the future to investigate the plasma-induced effects systematically.

Interestingly, previous studies on plasma treatment using light microscopy and, therefore, without the lice having been embedded in an agarose gel medium, revealed a different behaviour, including shrinkage of the samples over time. Moreover, a sudden rupture of the caecum or anterior midgut and an accompanying leakage of digested human blood into the thorax was observed in that study (see in Fig. 6). However, such rupture and leakage of blood was not observed in the present work using LSFM, possibly due to differences in sample preparation, mounting and imaging modalities. During the LSFM measurements in the present study, the lice were completely embedded in agarose gel; in contrast, light microscopy is carried out without any embedding. This difference leads to variations in plasma-induced reactions and mechanisms. However, LSFM represents a technique that allows the researcher to maintain the sample in a more stable condition and features a comparatively high-depth resolution. It has thus a high potential for the investigation of blood-sucking arthropods.

**Fig. 6:**
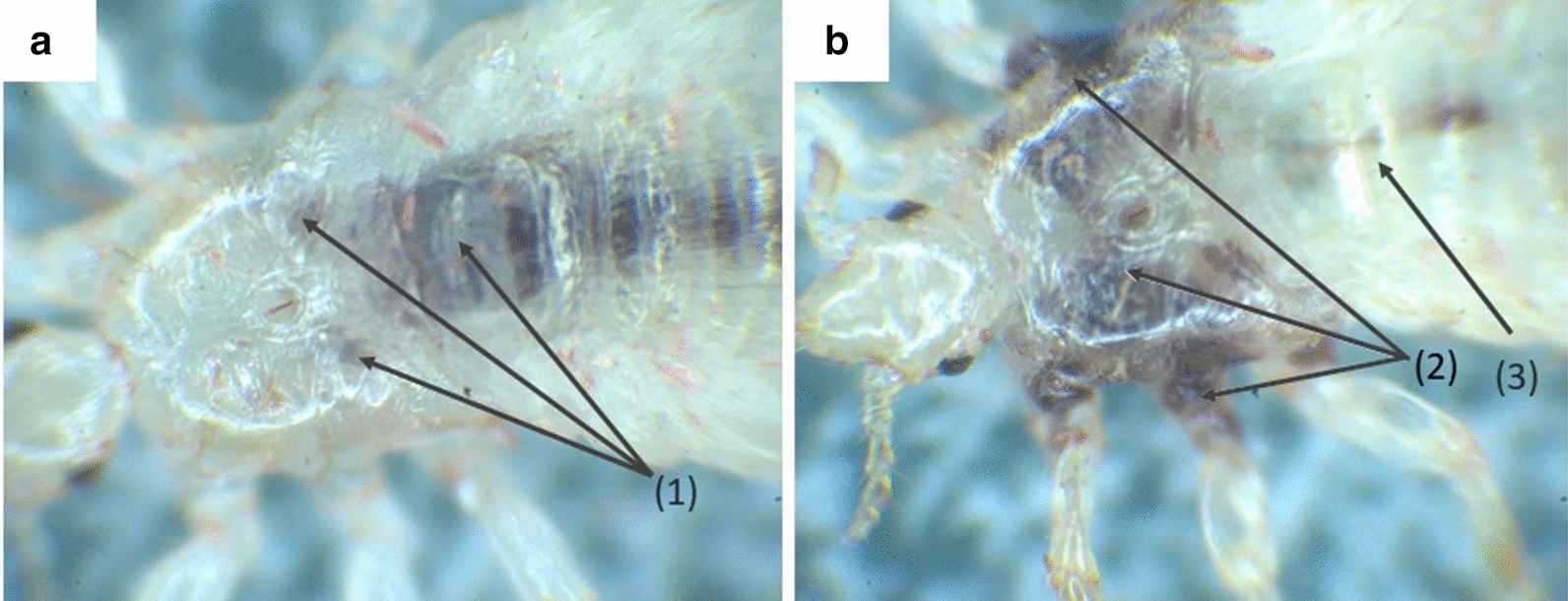
Light microscopy study of a louse subjected to plasma treatment. **a** Intact louse directly after treatment. Ingested blood is visible in the midgut and both caeca* (1)*. **b** Severely damaged louse several minutes after plasma treatment. The blood from the midgut* (3)* has leaked into the thorax region, successively entering the legs; the thorax of the louse is caved in, as are the extremities* (2)*

The results of this study showed that LSFM is suitable for 3D imaging of larger organisms, such as adult *Pediculus humanus humanus*. Staining of the samples via blood-feeding through an artificial membrane leads to a significant improvement in contrast and image quality, and allows for the identification and analysis of single organs. Thanks to the large field of view and mounting method, entire living organisms can be investigated, and long-term observations are possible. Depending on the anatomical characteristics of the investigated organisms, observation times can range in duration from several hours (*Pediculus humanus*) up to several days (*Danio rerio*). Further enhancement of the penetration depth and the contrast could be achieved by a rotation of the samples for a tomographic reconstruction. Moreover, a longer digestion interval between feeding/staining and scanning could contribute to an increased penetration depth. However, the degradation of dyes could also be an issue in this context, although there is no clear proof of degradation in the images obtained.

In combination with sample staining via membrane feeding, LSFM was shown to be a promising technique for the investigation of entire organisms, especially hematophagous arthropods that can be fed using in vitro feeding systems. In addition to lice fed in vitro [25, 28], these include, for example, *dipterans* such as mosquitoes, fleas, mites, argasid and ixodid ticks and bed bugs [29–33]. Since the LSFM technique recovers both the external and internal morphological structures of one organism longitudinally over time, it facilitates a more comprehensive analysis of the test animal and the observation of external and internal morphological changes. This could open the way for the application of new approaches for biological investigations and studies on toxicologically and non-toxicologically based effects or modes of action of products, as well as procedures to control arthropods of medical or veterinary importance. In fact, 3D-T-scans are of great interest for observing structural changes in the living organism, for example after applying insecticides or other remedies, and for analysing the accompanying modes of actions.

To summarise, the present work demonstrates that LSFM, in combination with the method of introducing a dye to a living organism by membrane feeding, could be a new technique for precise anatomic studies of bloodsucking insects or other specimens. Remarkably, the protocol allows imaging of in vivo samples, without the need for sample preparation required by histology or other ex vivo techniques. Deformations that could be induced by chemically clearing of a sample or its sectioning with a microtome are thus avoided. Moreover, the study of living samples provides the opportunity to analyse not only anatomical features but also functional characteristics of the animal. Apart from the observation of anatomical characteristics, i.e., inner organs, the morphology and thus the outer shape of bloodsucking arthropods can be visualised and examined, which enables evaluation of the impact of different pest treatment approaches and the study of their underlying mechanisms. Since an accelerated spread of pest arthropods is being promoted by advancing globalisation and climate change, such an expansion of sustainable pest control methods is of great interest. Against this background, further improvements of the method described here for measuring arthropods will be carried out in ongoing work. This includes, for example, testing different concentrations of the dye and assessing the performance of the technique in terms of illumination, imaging quality and contrast.

## Data Availability

The datasets used and/or analysed during the current study are available from the corresponding author on reasonable request.

## References

[1] Galland R, Grenci G, Aravind A, Viasnoff V, Studer V, Sibarita J-B (2015). 3D high- and super-resolution imaging using single-objective SPIM. Nat Methods.

[2] Pitrone PG, Schindelin J, Stuyvenberg L, Preibisch S, Weber M, Eliceiri KW (2013). OpenSPIM: an open-access light-sheet microscopy platform. Nat Methods.

[3] Candeo A, Doccula FG, Valentini G, Bassi A, Costa A (2017). Light sheet fluorescence microscopy quantifies calcium oscillations in root hairs of *Arabidopsis**thaliana*. Plant Cell Physiol.

[4] Aakhte M, Akhlaghi EA, Müller H-AJ (2018). SSPIM: a beam shaping toolbox for structured selective plane illumination microscopy. Sci Rep.

[5] Strobl F, Klees S, Stelzer EHK. Light sheet-based fluorescence microscopy of living or fixed and stained *Tribolium**castaneum* embryos. J Vis Exp. 2017;122:55629. 10.3791/55629. 10.3791/55629 PMC5565123 28518097

[6] Iachetta R, Ambrosone A, Klimovich A, Wittlieb J, Onorato G, Candeo A, et al. Real time dynamics of β-catenin expression during Hydra development, regeneration and Wnt signalling activation. Int J Dev Biol. 2018;62:311–8. 10.1387/ijdb.180092ct. 10.1387/ijdb.180092ct 29877570

[7] Bassi A, Schmid B, Huisken J (2015). Optical tomography complements light sheet microscopy for in toto imaging of zebrafish development. Development.

[8] Mikut R, Dickmeis T, Driever W, Geurts P, Hamprecht FA, Kausler BX (2013). Automated processing of zebrafish imaging data: a survey. Zebrafish.

[9] Lischik CQ, Adelmann L, Wittbrodt J (2019). Enhanced in vivo-imaging in medaka by optimized anaesthesia, fluorescent protein selection and removal of pigmentation. PLoS ONE.

[10] Strnad P, Gunther S, Reichmann J, Krzic U, Balazs B, de Medeiros G (2016). Inverted light-sheet microscope for imaging mouse pre-implantation development. Nat Methods.

[11] Kirkness EF, Haas BJ, Sun W, Braig HR, Perotti MA, Clark JM (2010). Genome sequences of the human body louse and its primary endosymbiont provide insights into the permanent parasitic lifestyle. Proc Natl Acad Sci USA.

[12] Toloza AC, Zygadlo J, Biurrun F, Rotman A, Picollo MI (2010). Bioactivity of Argentinean essential oils against permethrin-resistant head lice, *Pediculus**humanus**capitis*. J Insect Sci.

[13] Toloza AC, Laguna MF, Ortega-Insaurralde I, Vassena C, Risau-Gusman S (2018). Insights about head lice transmission from field data and mathematical modeling. J Med Entomol.

[14] Olds BP, Coates BS, Steele LD, Sun W, Agunbiade TA, Yoon KS (2012). Comparison of the transcriptional profiles of head and body lice. Insect Mol Biol.

[15] Veracx A, Raoult D (2012). Biology and genetics of human head and body lice. Trends Parasitol.

[16] Bressa MJ, Papeschi AG, Toloza AC (2015). Cytogenetic features of human head and body lice (Phthiraptera: Pediculidae). J Med Entomol.

[17] Tovar-Corona JM, Castillo-Morales A, Chen L, Olds BP, Clark JM, Reynolds SE (2015). Alternative splice in alternative lice. Mol Biol Evol.

[18] Boyd BM, Allen JM, Nguyen N-P, Vachaspati P, Quicksall ZS, Warnow T (2017). Primates, lice and bacteria: speciation and genome evolution in the symbionts of hominid lice. Mol Biol Evol.

[19] Ferris GF (1951). The sucking lice.

[20] Burgess IF. The mode of action of dimeticone 4% lotion against head lice, *Pediculus**capitis*. BMC Pharmacol. 2009;9:3. 10.1186/1471-2210-9-3. 10.1186/1471-2210-9-3 PMC2652450 19232080

[21] Ortega Insaurralde I, Minoli S, Toloza AC, Picollo MI, Barrozo RB (2019). The sensory machinery of the head louse *Pediculus**humanus**capitis*: from the antennae to the brain. Front Physiol.

[22] Greger K, Swoger J, Stelzer EHK (2007). Basic building units and properties of a fluorescence single plane illumination microscope. Rev Sci Instrum.

[23] Dodt H-U, Leischner U, Schierloh A, Jährling N, Mauch CP, Deininger K (2007). Ultramicroscopy: three-dimensional visualization of neuronal networks in the whole mouse brain. Nat Methods.

[24] Candeo A, Sana I, Ferrari E, Maiuri L, D'Andrea C, Valentini G (2016). Virtual unfolding of light sheet fluorescence microscopy dataset for quantitative analysis of the mouse intestine. J Biomed Opt.

[25] Habedank B, Schrader G, Scheurer S, Schein E. Investigations on the in vitro feeding and in vitro breeding of the human body louse *Pediculus**humanus**corporis* (Anoplura: Pediculidae). In: Robinson WH, Rettich F, Rambo GW, editors. Proceedings of the 3rd international conference on urban pests. Prague: Grafické závody; 1999. p. 241–8. (ISBN: 8023842579). https://www.icup.org.uk/media/lcylrz2z/icup423.pdf. Accessed 19 Jan 2022.

[26] ten Bosch L, Habedank B, Siebert D, Mrotzek J, Viöl W. Cold atmospheric pressure plasma comb—a physical approach for pediculosis treatment. Int J Environ Res Public Health. 2019;16(1):19. 10.3390/ijerph16010019. 10.3390/ijerph16010019 PMC6338894 30577656

[27] ten Bosch L, Habedank B, Siebert D (2020). Erratum: cold atmospheric pressure plasma comb—a physical approach for pediculosis treatment. Int J Environ Res Public Health..

[28] Takano-lee M, Yoon KS, Edman JD, Mullens BA, Clark JM (2003). In vivo and in vitro rearing of *Pediculus**humanus**capitis* (Anoplura: Pediculidae). J Med Entomol.

[29] Habedank B, Hiepe T, Montag C. Untersuchungen zur In-vitro-Fütterung von Zecken - Argasidae und Ixodidae. Mitt Österr Ges Tropenmed Parasitol. 1994;16:107–14. https://www.zobodat.at/pdf/MOGTP_16_0107-0114.pdf. Accessed 19 Jan 2022.

[30] Montes C, Cuadrillero C, Vilella D (2002). Maintenance of a laboratory colony of *Cimex lectularius* (Hemiptera: Cimicidae) using an artificial feeding technique. J Med Entomol.

[31] Wade SE, Georgi JR (1988). Survival and reproduction of artificially fed cat fleas, *Ctenocephalides**felis* Bouché (Siphonaptera: Pulicidae). J Med Entomol.

[32] Kröber T, Guerin PM (2007). In vitro feeding assays for hard ticks. Trends Parasitol.

[33] González J, Bickerton M, Toledo A (2021). Applications of artificial membrane feeding for ixodid ticks. Acta Trop.

